# Psoriasis Severity—A Risk Factor of Insulin Resistance Independent of Metabolic Syndrome

**DOI:** 10.3390/ijerph15071486

**Published:** 2018-07-13

**Authors:** Melita Vuksic Polic, Maja Miskulin, Martina Smolic, Kristina Kralik, Ivan Miskulin, Maja Cigrovski Berkovic, Ines Bilic Curcic

**Affiliations:** 1 Department of Dermatology, University Hospital Center Osijek, 31000 Osijek, Croatia; melyderma@gmail.com; 2 Faculty of Medicine Osijek, Josip Juraj Strossmayer University of Osijek, 31000 Osijek, Croatia; miskulin.maja@gmail.com (M.M.); martina.smolic@mefos.hr (M.S.); kristina.kralik@gmail.com (K.K.); ivan.miskulin@mefos.hr (I.M.); 3 Faculty of Dental Medicine and Health, Josip Juraj Strossmayer University of Osijek, Crkvena 21, 31000 Osijek, Croatia; 4 Department of Endocrinology, Diabetes and Metabolism, University Hospital Centre Sestre Milosrdnice, 10000 Zagreb, Croatia; maja.cigrovskiberkovic@gmail.com

**Keywords:** psoriasis severity, insulin resistance, chronic inflammation, cardiovascular risk factor

## Abstract

Background: It is still debatable whether psoriasis increases cardiovascular risk indirectly since it is associated with metabolic syndrome or is an independent cardiovascular risk factor. The aim of this study was to evaluate psoriasis severity as an independent predictor of insulin resistance (IR) irrespective of the presence of metabolic syndrome (MetS). Methods: This was a case control study including 128 patients stratified into two groups: patients with psoriasis and metabolic syndrome vs. patients with psoriasis and no metabolic syndrome. MetS was diagnosed according to ATP III criteria with homeostatic model assessment of insulin resistance (HOMA-IR), as well as a homeostatic model assessment of beta cell function (HOMA-β) were calculated. Results: Compared to subjects without metabolic syndrome, patients with metabolic syndrome had a significantly higher Psoriasis Area Severity Index (PASI) values (*p* < 0.001). The strongest correlation was established for HOMA-IR and the PASI index (*p* < 0.001), even after adjustment for body mass index (BMI) in regression analysis model. In patients without MetS and severe forms of disease, the HOMA-IR and HOMA-β values were significantly higher compared to mild forms of disease (*p* < 0.001 for all) while in subjects with MetS no difference was established for HOMA-IR or HOMA-β based on disease severity. Conclusions: Psoriasis severity is an independent risk factor of HOMA-IR, the strongest association being present in the non-MetS group, who still had preserved beta cell function suggesting direct promotion of atherosclerosis via insulin resistance depending on the disease severity, but irrespective of the presence of metabolic syndrome.

## 1. Introduction

Psoriasis vulgaris (PV) is a chronic recurrent inflammatory skin disease that occurs in genetically predisposed individuals, influenced by various endogenous and exogenous transducing factors [1]. The prevalence of psoriasis ranges from 0.91 in America to 8.5 in Norway [2]. Clinical manifestation is characterized by appearance of erythematosquamous papules or plaques of various sizes, which are typically symmetrically distributed over the knees, elbows, genital area, scalp and body [3]. There are different clinical forms of psoriasis and the most common is the chronic plaque psoriasis (psoriasis vulgaris) that occurs in about 80–85% of the patients. The clinical course is chronic, characterized by phases of remission and exacerbation [1]. It is believed that psoriasis is mediated by T-lymphocytes, as a result of impaired activation of both acquired and gained immunity [4]. Although in the past, it considered to be exclusively skin disease, psoriasis is now seen as a systemic disease with associated comorbidities [1,5]. The appearance of comorbidity correlates with the severity of the clinical presentation, and their number usually increases with age and disease duration [6]. One of the most common and most important comorbidities is metabolic syndrome, encompassing risk factors important for development of cardiovascular diseases: hypertension, obesity, insulin resistance, and dyslipidemia [7,8]. Patients with psoriasis have a higher incidence of metabolic syndrome than general population, and patients with a more severe form of psoriasis have a higher incidence of metabolic syndrome than those with a mild form [9]. Insulin resistance is a central disturbance common not only to obesity, metabolic syndrome, and type 2 diabetes, but also to cardiovascular diseases [10]. This is a condition where normal insulin levels are not sufficient for adequate insulin response to fat, muscle tissue, and liver cells [11]. Inflammatory cytokines involved in development of insulin resistance, such as TNF-α, IL-6 together with leptin and adiponectin, were also found in psoriasis [12,13]. Furthermore, insulin resistance was found in lean patients with psoriasis [14], while psoriasis is an independent risk factor for development of diabetes type 2 [15,16]. There is still a controversy about psoriatic inflammation driving cardiovascular comorbidity via insulin resistance independent of other traditional components of metabolic syndrome [17]. This is perceived as especially important, and according to the recently published algorithm, patients with psoriasis should be more closely monitored in the presence of metabolic syndrome [18]. The aim of this study was to evaluate importance of psoriasis as an independent predictor of insulin resistance irrespective of the presence of metabolic syndrome and to evaluate quality of life in psoriasis patients in relation to metabolic syndrome.

## 2. Materials and Methods

This was a case control study including 128 patients with psoriasis; of which 64 with and 64 without metabolic syndrome. The study has been assessed and approved by the Ethics Committee of Osijek University Hospital (R2: 22512-5/2015) and the Ethics Committee of Faculty of Medicine Osijek (2158-61-07-16-04). Informed consent was obtained from all patients included in the study. The study was conducted in accordance with the Declaration of Helsinki (2004).

The inclusion criteria were as follows: the patients were between 18–90 years of age and had pathohistological confirmation of psoriasis vulgaris diagnosis. Exclusion criteria were: drugs that can cause psoriasis (beta blockers, lithium, systemic antimalarial drugs), other forms of psoriasis (pustular, droplet, erythrodermic, inverse), other autoimmune diseases (data obtained through medical history questionnaire), systemic therapy (retinoids, methotrexate, biological therapy, corticosteroids), and patients with active liver disease.

Psoriasis vulgaris was diagnosed by clinical examination and pathohistological findings. Severity of disease was determined with the Psoriasis Area Severity Index (PASI index). The PASI index values ranged from 0 to 72. Mild psoriasis had a PASI index values up to 10, moderate to severe psoriasis had a PASI index of 10–20 and PASI index above 20 was defined as severe psoriasis. The Dermatology Life Quality Index (DLQI) values ranged from 0 to 30. The DLQI value higher than 10 implied poor quality of life. According to ATP III criteria [11] patients were categorized as those with and without metabolic syndrome. At study entry blood sugar (BG), cholesterol, triglycerides, HDL-cholesterol, LDL-cholesterol and uric acid levels were determined using routine laboratory assays, body mass index-BMI was calculated, and waist circumference and two sequential blood pressure measures were obtained. Homeostatic Model Assessment of Insulin Resistance (HOMA-IR), as well as Homeostatic Model Assessment of beta cell function (HOMA-β) were calculated according to formula: HOMA-IR = glucose (mmol/L) × insulin (mIU/L)/22.5; HOMA-β = 20 × insulin (mIU/L)/(glucose − 3.5)%).

All subjects completed an anonymous survey questionnaire. The questionnaire included demographic data (age, gender, place of permanent residence, education), body height and weight, medical history related to psoriasis, comorbidities, medical history on other skin diseases and other chronic autoimmune diseases, data on daily medication therapy, the assessment of daily stress exposure through a five-stage Likert scale and information on lifestyle habits involving smoking and consumption of alcohol.

### Statistical Methods

Categorical data are represented by absolute and relative frequencies. Numerical data are described by arithmetic mean and standard deviation in case of distribution following normal and in other cases median and interquartile range boundaries. The variance of the category variables was tested by the c2 test, as well as by Fisher’s exact test. The normality of the distribution of numeric variables was tested by Kolmogorov–Smirnov test. The differences of normal distribution of numerical variables between the two independent groups (metabolic and nonmetabolic syndrome patients) were tested by the Student’s *t*-test, and in the case of a deviation from the normal distribution by Mann-Whitney’s in-test. The differences between the normally distributed numeric variables in case of 3 and more independent groups were tested by variance analysis (ANOVA) and in case of a deviation from the normal distribution by the Kruskal-Wallis test. The correlation of normally distributed numeric variables was evaluated by the Pearson coefficient of correlation r and in case of a deviation from the normal distribution by Spearman’s coefficient of correlation ρ (rho). Regression analysis determined which of the predictors affected the development of metabolic syndrome in patients with psoriasis vulgaris the most. All *p* values are two-sided. The level of significance is set at a = 0.05. Statistical analysis was performed by the statistical program SPSS (version 16.0. SPSS Inc., Chicago, IL, USA).

## 3. Results

### 3.1. Baseline Characteristics of Subjects and Comorbidities

Baseline characteristics of subjects are summarized in Table 1. The study included 128 patients, 64 (50%) with and 64 (50%) without metabolic syndrome. The average age of the participants was 55, with the corresponding interquartile range of 42 to 65 years. The average age at the onset of the disease was 33 (interquartile range from 20 to 50 years), with no significant differences between the groups.

Nonmetabolic syndrome group had higher values of HDL (*p* < 0.001), while subjects with metabolic syndrome were more often obese (*p* = 0.02), and had higher BMI, waist circumference, and systolic and diastolic blood pressure values (*p* < 0.001 for all). Also, HOMA-IR (*p* = 0.008), BG values (*p* = 0.004) and HbA1c (*p* = 0.02) were significantly higher in patients with metabolic syndrome. Furthermore, patients with metabolic syndrome had higher values of triglycerides (*p* = 0.01), LDL (*p* < 0.001) and uric acid (*p* = 0.02), while PASI index values were lower in subjects without metabolic syndrome (*p* < 0.001). Patients in both groups had good glycemic control with HbA1c below 6% as well as diastolic blood pressure (below 90 mmHg), and systolic blood pressure (below 140 mmHg) (Table 1). There was no statistical significance in alcohol consumption and smoking between groups. Patients with metabolic syndrome had a poorer quality of life compared to nonmetabolic syndrome group (*p* = 0.01).

Hypertension was the most common concomitant disease occurring in 47 (36.7%) patients, followed by hyperlipidemia in 34 (26.6%), diabetes type 2 in 17 (13.3%) and depression and other psychiatric illness in 14 (10.9%) participants, with no significant differences between the groups. Only 14 (17.1%) patients had another chronic illness (diabetes, hyperlipidemia, hypertension) prior to the onset of psoriasis, while 68 (82.9%) had a second chronic illness following psoriasis. At study entry out of 128 patients, 37 (28.9%) had newly diagnosed metabolic syndrome, while 108 (84.37%) had some kind of metabolic disturbance including lipid disorders, increased urate levels, hypertension, or insulin resistance.

### 3.2. Dose Effect of Psoriasis on the Specific Components of Metabolic Syndrome

Significant positive correlation with the PASI index was established for systolic blood pressure, HBA1c, insulin and C peptide, LDL, uric acid, HOMA-IR, and HOMA-β (Table 2). The strongest correlation was determined between HOMA-IR and the PASI index (*p* < 0.001), even after adjustment for BMI (F = 4.13 *p* = 0.02) in the regression analysis model. In patients without metabolic syndrome, higher values of HOMA-IR (*p* = 0.004) and HOMA-β (*p* = 0.007) had patients with moderate to severe and severe form of the disease. In patients with metabolic syndrome there was no difference between HOMA-IR or HOMA-β based on the severity of the disease (Table 3).

## 4. Discussion

There is a growing body of evidence, particularly epidemiological data, supporting the link between psoriasis and cardiovascular comorbidity [19,20]. Possible explanation could lay in the idea that psoriasis is not an isolated cutaneous inflammation, but rather a chronic systemic inflammatory disease triggering other comorbid conditions [21]. The same was observed in our study, since the vast majority of patients (68%) developed a second chronic illness following psoriasis vulgaris, irrespective of the presence of metabolic syndrome. Insulin resistance, defined as decreased sensitivity to metabolic actions of insulin, is important component of cardiovascular disorders, through decreased production of endothelial nitric-oxide-induced endothelial dysfunction. It is aggravated by the presence of TNF-α, a key proinflammatory cytokine in the pathogenesis of psoriasis [22], and vice versa it can lead to unfavorable long term prognosis of psoriasis patient [23]. The presented results confirmed that insulin resistance influenced psoriasis severity since subjects with psoriasis and metabolic syndrome had higher PASI values than those with no metabolic syndrome. This is also consistent with the results of a large meta-analysis of observational studies in which psoriasis patients had a higher prevalence of metabolic syndrome compared with the general population, and patients with more severe psoriasis had greater odds of metabolic syndrome than those with milder psoriasis [9]. This so-called “dose effect” of psoriasis on the metabolic syndrome could also be applied to cardiovascular risk which remains even after adjusting for major cardio-vascular risk factors, such as hypertension, diabetes mellitus, and hyperlipidemia [24] implicating psoriatic inflammation as a sole culprit in CV disease independent of other traditional risk factors. This could be explained by the concept of “psoriatic march” regarding psoriasis as a chronic systemic inflammatory disorder, with not only the classical markers for systemic inflammation, but also elevated resistin and leptin levels [22,25]. Those could trigger the onset of insulin resistance, a known factor of atherosclerosis [26]. However, the question is whether concomitant low grade inflammation present in the metabolic syndrome is necessary to instigate development of insulin resistance in psoriatic patients? In other words, could metabolically healthy individuals develop insulin resistance in a dose-dependent manner with regard to psoriasis severity? In our study, the strongest positive correlation existed between the PASI index and HOMA-IR, which persisted even after adjustment for BMI. Quite interestingly, no correlation between BMI and waist circumference with psoriasis severity was demonstrated indicating that insulin resistance was associated with clinical presentation of psoriasis irrespective of body weight. In order to confirm this theory, we have investigated HOMA-IR and HOMA-β in both groups of patients in relation to psoriasis severity and it has been determined that significantly higher values of HOMA-IR were found in patients with moderate to severe form of psoriasis without MetS, while no difference was established in patients with MetS. One of the possible explanations is that in nonobese individuals high levels of proinflammatory cytokines found in severe forms of psoriasis are responsible for development of insulin resistance through modification of the insulin signaling pathway. On the other hand, HOMA-β values were higher in that particular group of patients, indicating an early compensatory mechanism of the beta cell, whose function was still preserved in the absence of MetS, whereas there was no difference between HOMA-IR and HOMA-β in those with already developed MetS.

This study has many strengths. Compared to previous studies [27,28,29], retrospectively using administrative databases and estimating the severity of psoriasis as required for systematic therapy usage, in this study, severity of the disease was evaluated accurately by using a validated clinical index. Furthermore, the exclusion of patients treated with systemic drugs (e.g., acitretin, methotrexate, cyclosporine) minimized the possibility of drug effect on the appearance of MetS.

Limitations of this study include relatively small number of participants and a cross-sectional design. In the future, longitudinal prospective studies are needed with larger number of participants, including those that were treated with anti-inflammatory systemic drugs, which would give us a clear insight on the impact of treatment of severe form of psoriasis on development of insulin resistance and diabetes type 2.

## 5. Conclusions

We can conclude that psoriasis severity is an independent predictor of HOMA-IR, the strongest association being present in the nonmetabolic syndrome group, who still had preserved beta cell function. Our results may help during the initial screening of patients who do not have all the criteria for diagnosis of the metabolic syndrome but are in need of adequate and well-timed monitoring by both dermatologists and specialists in internal medicine (cardiologists, diabetologists, rheumatologists), in order to be treated by introduction of adequate drugs, which would prevent occurrence of the metabolic syndrome, type 2 diabetes, and finally cardiovascular disease.

## Figures and Tables

**Table 1:** Baseline characteristics of patients according to groups.

Variable	Median (Interquartile Range)			*p* *
	No Metabolic Syndrome	With Metabolic Syndrome	Total	
Average age (years)	55 (43–65)	55 (42–65)	55 (42–65)	NS
Average age at diagnosis (years)	38 (24–53)	30 (20–50)	33 (20–50)	NS
Body mass index (kg/m^2^)	26.1 (23.4–29.7)	30.1 (26.8–32.9)	27.9 (24.8–31.6)	<0.001
Waist circumference (cm)	95 (90–100)	103 (99–109)	100 (91–106)	<0.001
Systolic blood pressure (mmHg)	120 (120–130)	140 (120–140)	129 (120–140)	<0.001
Diastolic blood pressure (mmHg)	80 (75–80)	85 (80–90)	80 (80–90)	<0.001
FBG (mmoL/L)	5.5 (5–6)	6.5 (5–7)	5.7 (5–6.6)	0.004
HbA1c (%)	5.3 (4.9–5.8)	5.6 (5.2–6)	5.5 (5.1–5.9)	0.02
Insulin (pmoL/L)	9.2 (5–16.6)	12.6 (8.6–16.9)	11.2 (5.7–16.7)	NS
C-Peptide (mmoL/L)	1.1 (0.8–1.3)	1.1 (0.8–1.3)	1.1 (0.8–1.3)	NS
Cholesterol (mmoL/L)	5.5 (4.7–6)	5.8 (5.2–6.6)	5.5 (4.8–6.3)	NS
Triglycerides (mmoL/L)	1.3 (1.1–1.8)	1.8 (1.3–2.4)	1.6 (1.2–2.2)	0.01
HDL (mmoL/L)	1.3 (1.2–1.5)	1.1 (1.1–1.2)	1.2 (1.1–1.4)	<0.001
LDL (mmoL/L)	3.2 (2.9–3.5)	3.6 (3.4–4.1)	3.4 (3–3.8)	<0.001
Uric acid (mmoL/L)	292 (270–320)	310.5 (278–368.3)	295 (273.5–346)	0.02
HOMA-IR	2.23 (1.29–3.76)	3.40 (2.05–5.16)	2.76 (1.57–4.56)	0.008
HOMA-β	93.46 (45.15–160.27)	95.16 (58.83–214.68)	93.47 (51.66–198.98)	NS
PASI index	7.8 (5–18)	21.4 (15–28.5)	17 (7–25.2)	<0.001
Smoking (*n*)	61	64	125	NS
Alcohol consumption (*n*)	59	61	120	NS
DLQI index	6 (2–13)	11 (5–17)	8 (3.75–15)	0.01

* Mann-Whitney U test; NS, not significant.

**Table 2:** Correlation of disease severity and components of metabolic syndrome.

Spearman’s Correlation Coefficient for PASI Index		
Variable	ρ (Rho)	*p* Value
Body weight	0.082	NS
Body height	0.058	NS
Waist circumference	0.181	NS
Systolic blood pressure	0.218	0.01
Diastolic blood pressure	0.165	NS
FBG	0.095	NS
HbA1c	0.192	0.04
Insulin	0.299	0.001
C peptide	0.169	NS
Cholesterol	−0.009	NS
Triglycerides	0.038	NS
HDL	−0.101	NS
LDL	0.236	0.008
Uric acid	0.269	0.003
HOMA-IR	0.320	<0.001
HOMA-β	0.208	0.02

NS, not significant.

**Table 3:** HOMA-IR and HOMA-β according to disease severity.

Variable	Median (Interquartile Range)			*p* *
	Mild Form	Moderate to Severe Form	Severe Form	
**All Subjects**				
HOMA-IR	1.8 (1.2–3.1)	3.6 (2.1–5.3)	3.5 (2.2–5.1)	<0.001
HOMA-β	65.7 (39.3–108.2)	110.4 (52.2–251.7)	120 (67.9–215.4)	0.008
**No Metabolic Syndrome**				
HOMA-IR	1.9 (1.1–3.1)	3.1 (1.2–4.6)	4.1 (3–5.5)	0.004
HOMA-β	70.9 (39.9–110.2)	113.8 (58.8–230.6)	155.9 (103.3–417.8)	0.007
**With Metabolic Syndrome**				
HOMA-IR	1.8 (1.3–4.4)	4 (2.2–5.9)	2.8 (2–5)	NS
HOMA-β	51.5 (30–80)	107.1 (47.2–255.8)	90.4 (67.3–204.9)	NS

* Kruskal-Wallis test; NS, not significant.
